# The Role of Cardiolipin in Mitochondrial Function and Neurodegenerative Diseases

**DOI:** 10.3390/cells13070609

**Published:** 2024-03-30

**Authors:** José M. Fuentes, Patricia Morcillo

**Affiliations:** 1 Departamento de Bioquímica y Biología Molecular y Genética, Facultad de Enfermería y Terapia Ocupacional, Universidad de Extremadura, 10003 Cáceres, Spain; 2 Centro de Investigación Biomédica en Red en Enfermedades Neurodegenerativas, Instituto de Salud Carlos III (CIBER-CIBERNED-ISCIII), 28029 Madrid, Spain; 3 Instituto Universitario de Investigación Biosanitaria de Extremadura (INUBE), 10003 Cáceres, Spain; 4 Departmentof Neurology, Columbia University, New York, NY 10032, USA

**Keywords:** cardiolipin, mitochondria, biological functions, neurodegenerative diseases, therapeutic applications

## Abstract

Cardiolipin (CL) is a mitochondria-exclusive phospholipid synthesized in the inner mitochondrial membrane. CL plays a key role in mitochondrial membranes, impacting a plethora of functions this organelle performs. Consequently, it is conceivable that abnormalities in the CL content, composition, and level of oxidation may negatively impact mitochondrial function and dynamics, with important implications in a variety of diseases. This review concentrates on papers published in recent years, combined with basic and underexplored research in CL. We capture new findings on its biological functions in the mitochondria, as well as its association with neurodegenerative diseases such as Alzheimer’s disease or Parkinson’s disease. Lastly, we explore the potential applications of CL as a biomarker and pharmacological target to mitigate mitochondrial dysfunction.

## 1. Introduction 

**Figure 1:**
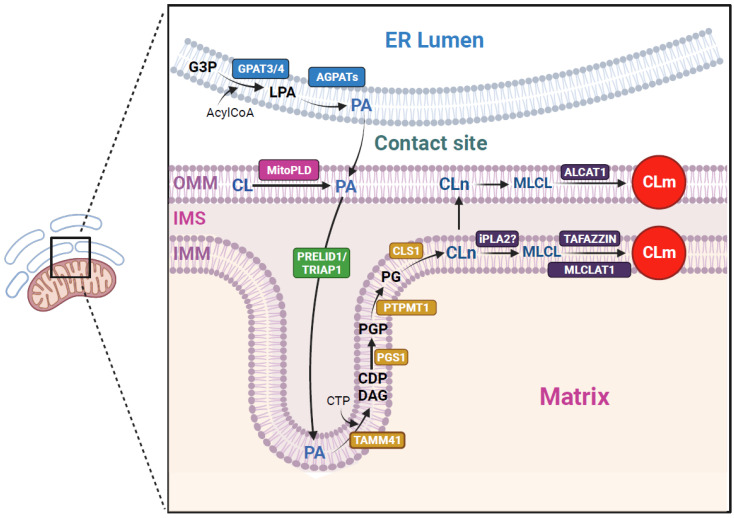
Schematic representation of CL biosynthesis and remodeling in mammals. CL production begins with PA, which can be sourced from several pathways where the enzymes GTATs and/or mitoPLD are present. PA needs to be transported to the IMM, where CL biosynthetic enzymes reside. PA shuttling from the OMM to the IMM is performed by PRELID1-TRIAP1. Inside of the IMM, PA is converted to CLn in a series of reactions catalyzed by several enzymes. Finally, CLn suffers a remodeling to become CLm in the IMM or OMM. Figure created with BioRender.com (accessed on 1 March 2024). Abbreviations: G3P, glycerol-3-phosphate, GPATs, G3P acyltransferases, AGPATs, LPA acyltransferases; PA, phosphatidic acid; CL, cardiolipin; PRELID1, protein of relevant evolutionary and lymphoid interest domain; TRIAP1, TP53-regulated inhibitor of apoptosis 1; TAM41, translocator assembly and maintenance homolog; PGS1, phosphatidylglycerol phosphate synthase; PTPMT1, protein-tyrosine phosphatase mitochondrial 1; CLS1, CL synthase; CLn, nascent CL; CLm, mature CL, iPLA2, phospholipase A2; MLCL, monolysocardiolipin; TAFAZZIN, tafazzin phospholipid remodeling enzyme; ALCAT1, acyl-CoA:lysocardiolipin acyltransferase 1; MLCLAT1, acyl-CoA:monolysocardiolipin acyltransferase 1.

Cardiolipin (CL; 1,3-bis(sn-3′-phosphatidyl)-sn-glycerol) was isolated from beef hearts by Mary C. Pangborn in the 1940s [1]. It is predominantly localized and synthesized in the inner mitochondrial membrane (IMM) and constitutes approximately 15–20% of the total mitochondrial phospholipids [2]. It has a conical shape characterized by a double glycerophosphate backbone, three glycerol moieties, and four fatty acyl (FA) chains (rather than two fatty acyl side chains typically observed in phospholipid structures) which can show different lengths or degrees of saturation. It exerts lateral pressure within the membrane causing negative membrane curvature [3]. De novo CL biosynthesis in mammals is a multi-step process that begins with the synthesis and transport of phosphatidic acid (PA) from the endoplasmic reticulum (ER) to mitochondria. Alternatively, mitochondrial phospholipase D (MitoPLD) can hydrolyze CL to generate PA in the OMM [4]. Next, PA is translocated to the IMM where several enzymes are involved in CL biosynthesis, including TAM41 mitochondrial translocator assembly and maintenance homolog (TAMM41); phosphatidylglycerol phosphate synthase (PGS1); protein-tyrosine phosphatase mitochondrial 1 (PTPMT1); and CL synthase (CLS1), which catalyzes the formation of nascent CL (CLn). CLn, which is characterized by the presence of saturated acyl chains, is remodeled by phospholipase A2 (PLA2) into the transient intermediate phospholipid monolysocardiolipin (MLCL), which is subsequently re-acylated by TAFAZZIN (tafazzin phospholipid remodeling enzyme) to form mature CL (CLm), which is enriched in unsaturated FA chains. Furthermore, CL remodeling can also occur via the acyl transferases ALCAT1 or MLCLAT1 [5]. However, the extent to which these alternative pathways contribute to CL remodeling is still under debate. A schematic representation of the synthesis and remodeling of CL is depicted in Figure 1.

CL exhibits the greatest structural diversity among all phospholipid classes due to its tetra-acylated structure. Notably, the arrangement of fatty acyl chains in CL is not stochastic. Each CL contains unique lengths or degrees of unsaturation in its fatty acid (FA) tails through post-synthetic remodeling, which appears to be tissue- and cell-specific. For instance, in the heart, the composition of CL is restricted to one dominant FA, the linoleic acid (18:2), resulting in a homogeneous population, whereas, in the brain, oleic acid (18:1) along with longer-chain unsaturated FA such as arachidonic acid (20:4) and docosahexaenoic acid (22:6) are the predominant side chains [6,7].

Neurodegenerative diseases affect millions of people globally, and, currently, treatments are very limited. While many of these diseases involve the misfolding and aggregation of proteins, leading to the formation of insoluble fibrils, tangles, and plaques, these findings alone almost certainly cannot explain the clinical diversity observed in these disorders. Intriguingly, mitochondrial dysfunction is strongly implicated in the pathogenesis of numerous neurodegenerative diseases such as Alzheimer’s disease (AD) [8], or Parkinson’s disease (PD) [9]. However, in most cases, how aberrant mitochondrial function leads to disease and phenotypes is unclear. Remarkably, recent studies have shown that alterations in mitochondrial homeostasis associated with neurodegeneration are influenced by mitochondrial lipid composition and overall lipid metabolism [10]. For this reason, there is a growing interest in the use of lipids as potential biomarkers for neurodegenerative diseases. Specifically, quantitative and structural changes in CL have been shown in the context of neurodegenerative diseases [11,12,13]. The identification of CL as a candidate biomarker in diagnosing neurodegenerative diseases has been supported by recent advancements in lipidomics studies using human blood from patients and animal models [14,15,16]. While the role of CL in mitochondria has been discussed in other reviews [17,18], here, we focus on new discoveries that appeared in recent years, coupled with basic and underexplored research in CL. Specifically, we explore new biological functions and the proteins affected by changes in CL content, leading to mitochondrial dysfunction. We discuss the role of CL in the pathology of neurodegenerative disorders and its use as a biomarker. Finally, we address the discovery of recent CL-binders and their potential role as therapeutic agents to ameliorate mitochondrial dysfunction and prevent neurodegeneration.

## 2. Physicochemical Properties and Detection of CL

The structure of CL differs from other phospholipids because two phosphatidates bind to a single central glycerol headgroup, resulting in a conical shape with a smaller cross-sectional area in the headgroup which restricts its mobility to interact with other phospholipid head groups. As a cone shape, with the polar region at the top and the flexible acyl chains at the base of the “cone”, it aggregates in structures with a negative curvature in the IMM facing the cristae structures. On the contrary, the positively curved outer monolayer leaflet primarily consists of phosphatidylcholine. Besides its preference for forming a non-bilayer structure, such as the hexagonal phase, CL can also adopt a bilayer structure with a preference for the lamellar phase in the presence of divalent cations [19,20]. In addition, the distribution of CL is determined by the lipid-scaffolding proteins prohibitin-1 and prohibitin-2, which may facilitate the remodeling process as part of the biosynthetic machinery [21].

**Figure 2:**
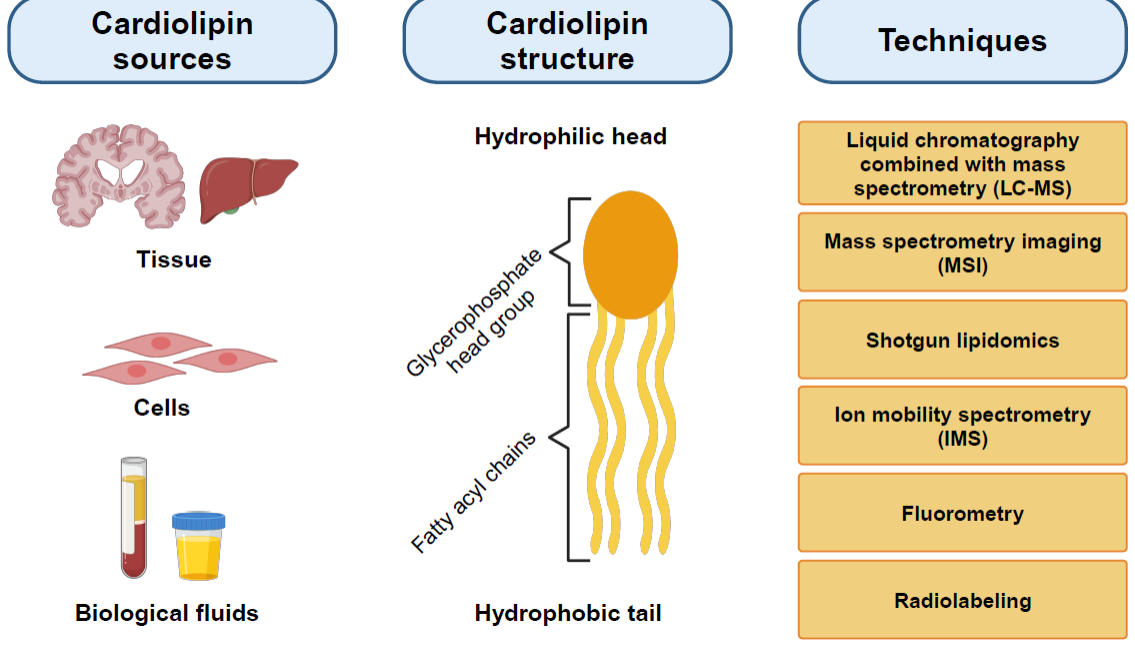
Schematic representation of CL sources (tissues, cells, and fluids), CL structure, and techniques for its detection.

The detection and quantification of CL constitute valuable tools for verifying mitochondrial dysfunction, characterizing the pathophysiological mechanisms of disease, and facilitating clinical diagnostics. Several methods are commonly used to analyze CL, mainly liquid chromatography combined with mass spectrometry (LC-MS), mass spectrometry imaging (MSI), shotgun lipidomics, ion mobility spectrometry (IMS), fluorometry, and radiolabeling (Figure 2). Before the analysis, it is necessary to extract and isolate the lipid content, unless fluorescent dyes such as 10-N-Nonyl acridine orange (NAO) are utilized, which can directly stain cells or tissues [22]. Mass spectrometric techniques are increasingly accessible and offer numerous advantages, particularly in sensitivity and specificity, over older chromatographic and fluorometric methods. In recent years, LC-MS-based multi-analyte/lipidomic assays have also been utilized in clinical practice.

Different biological samples can be employed in investigating the role of CL in health and disease. Samples include tissues such as brain, liver, or heart, and cell lines such as patient-derived fibroblasts, as well as biological fluids such as serum, urine, or cerebrospinal fluid. Moreover, induced pluripotent stem cells differentiated into disease-relevant cell types, including cortical neurons, myotubes, or cardiomyocytes, enable researchers to characterize CL in tissue-specific human disease models [23,24]. The specific samples studied in each disease are detailed in Section 5. Given the unavailability of brain biopsy samples from living patients, samples collected from blood, urine, or saliva can be used as an alternative to identify “CL signatures” capable of predicting the severity of the disease.

## 3. The Role of Cardiolipin in Mitochondrial Biological Functions

The structure features of CL allow it to play a crucial role in various mitochondrial processes, including the membrane architecture, the stabilization of the mitochondrial respiratory chain, the assembly of F_1_-F_0_ ATP synthase, the organization of supercomplexes, the regulation of mitochondrial dynamics, and ER–mitochondrial contact sites. Next, we describe new findings about the role of CL in mitochondrial biological functions. 

### 3.1. The Role of Cardiolipin in Mitochondrial Membrane Integrity

The structural and dynamic characteristics of mitochondrial membranes are significantly influenced by the biophysical properties of CL. An elevated CL concentration increases the lateral diffusion of lipids, affecting the mitochondria membrane composition [25]. The increased dipole potential observed in the IMM can be attributed to its elevated CL content in contrast to the outer mitochondrial membrane (OMM) [26]. Thus, CL may play a crucial role in controlling the flux rate of protons and ions during ATP production. Additionally, CL, characterized by a cone-like shape, accumulates in curved regions of the membrane, with higher sorting to more highly curved and negative membranes [3]. Apart from the biophysical effect of CL on mitochondrial membranes, CL is crucial for the formation and maintenance of the mitochondrial tubular cristae. Although the molecular mechanisms shaping cristae are becoming understood, the synergic contribution of three major protein complexes, OPA1, F_1_-F_0_ ATP synthase, and the MICOS complex, are recognized as essential for the formation and maintenance of cristae in eukaryotic cells [27]. It is well established that the cristae-forming protein complexes, F_1_-F_0_ ATP synthase, and the MICOS complex are stabilized by CL, which promotes cristae formation [28,29]. Specifically, CL binds to the subunit MIC27, which is part of the MIC10 subcomplex, preserving the integrity of the MICOS complex at the cristae [29,30,31,32]. Moreover, CL regulates the ATP synthase activity [33,34] (see next section for more details).

### 3.2. Role of Cardiolipin in Bioenergetics

CL is thought to act as a glue holding the respiratory supercomplex (RSC), high-molecular-weight structures consisting of complexes I, III, and IV in mammals [35]. RSCs are proposed to assist in the electron transfer between individual respiratory chain components acting to lessen oxidative damage [36]. Thus, CL plays an important role in the integrity of the RSC, reducing ROS generation [24]. CL also plays a role in anchoring cytochrome c to the IMM and facilitates the electron transfer from complex III to complex IV [37]. In addition, CL supports efficient OXPHOS activity. Recent cryogenic electron microscopy studies in bovine mitochondria have also revealed that CL binds tightly to F_1_-F_0_ ATP synthase and stabilizes its interaction with the IMM, thus controlling proton leakage and improving ATP generation, in addition to participating in dimer assembly [34]. Additionally, theoretical simulations show that the transient binding of CL to F_1_-F_0_ ATP synthase lubricates its rotor [33]. Finally, a recent study has shown that the mitochondrial ribosome binds CL, thereby stabilizing the IMM association of the protein translation machinery and supporting the biogenesis of mitochondrial OXPHOS proteins [38].

### 3.3. Role of Cardiolipin in Mitochondrial Protein Translocation

CL is also indispensable for the stability, assembly, and activity of mitochondrial carrier proteins and ion channels. Specifically, it plays a crucial role in shaping the conformation and assembly of the ADP/ATP carrier protein (AAC) which resides in the IMM and exchanges ADP and ATP to enable OXPHOS [39,40]. CL is also vital for the assembly and functioning of the mitochondrial Ca^2+^ uniporter (MCU) complex, which co-ordinates cellular energy demands with mitochondrial bioenergetics through Ca^2+^ signaling [41]. CryoEM structures of the MCU holo-complex demonstrate that MCU tetramers are stabilized by eight CL molecules [42,43]. CL is also necessary for activating various mitochondrial proteins, such as pyruvate dehydrogenase [44], and frataxin, which are crucial for the biogenesis of mitochondrial iron–sulfur clusters [45].

### 3.4. Role of Cardiolipin in Mitochondrial Dynamics

Mitochondria are dynamic organelles orchestrating continuous cycles of fusion and division, crucial for regulating mitochondrial size, number, distribution, function, and turnover [46]. CL plays a role in the maintenance of mitochondrial dynamics by regulating the fusion and fission machinery. It is known that CL interacts with DRP1 (dynamin-related protein 1) promoting mitochondrial constriction [47]. Conversely, it has been shown that the externalization of CL in the outer leaflet of the IMM plays an important role in Opa1-mediated efficiency fusion [48]. Human OPA1 is embedded into CL-containing membranes through a lipid-binding paddle domain [49].

### 3.5. Role of Cardiolipin in ER–Mitochondrial Contact Sites

The communication between the ER and mitochondria takes place through their networks, both physically and functionally [50]. These interactions, referred to as mitochondria-associated ER membranes (MAMs), play a crucial role in transporting lipids from the ER, the primary site of lipid biosynthesis, to the mitochondria [51]. New findings indicate that CL plays a role in regulating ER–mitochondrial contact sites. First, a recent study has shown that the tyrosine phosphatase-interacting protein 5 (1PTPIP51) binds and transfers PA from the ER to mitochondria at MAM, and its deletion resulted in reduced mitochondrial CL levels [52]. PTPIP51 also interacts with VAPB (vesicle-associated membrane protein-associated protein B), an ER protein, to form a tether between the ER and the mitochondria [52]. Second, both the enzyme ALCAT1 and the lipid CL have been found to be enriched at MAM [53]. Third, MICOS subcomplexes assemble in proximity to ER contact sites [54]. Together, these studies provide evidence that CL metabolism may be modulated at ER–mitochondrial contact sites, thereby influencing their function. Consequently, disruptions in ER–mitochondrial connectivity may directly contribute to mitochondrial ultrastructural disorganization, leading to disorders associated with CL homeostasis.

### 3.6. Other Functions

Recently, the discovery of new interactions between CL and other proteins suggests that CL is involved in additional activities. For instance, CL showed extremely higher affinity for the potassium channel KcsA (K^+^ channel, *Streptomyces lividans* A) than for monoanionic lipids [55]. Furthermore, CL regulates the activity of the mitochondrial ABC transporter 10 (ABCB10), which is an exporter localized in the IMM [56].

## 4. The Role of Cardiolipin Alterations in Mitochondrial Dysfunction

Pathological alterations in CL profiles have been reported in several diseases, including loss of content, aberrant remodeling, and/or peroxidation. The reduction in CL content may result from increased CL degradation catalyzed by calcium-independent phospholipase A2γ (iPLA2γ) [57]. Alterations/mutations in enzymes responsible for CL biosynthesis or remodeling lead to aberrant CL [58]. For instance, mutations in TAFAZZIN lead to CL deficiency in the cardiomyopathy Barth syndrome [59]. Additionally, CL is highly susceptible to oxidation due to its elevated content of unsaturated fatty acids, mainly linoleic acid, and its proximity to the electron transport chain (ETC), the site of reactive oxygen species (ROS) production [60]. Under physiological conditions, approximately 15–20% of cytochrome c binds to CL in the IMM [61]. CL can translocate to the OMM when ROS are present, thereby effectively increasing the likelihood of CL binding to cytochrome c. Subsequently, cytochrome c, which normally acts as a shuttle of electrons between respiratory complexes III and IV, becomes a CL peroxidase, capable of catalyzing CL oxidation and peroxidation reactions [62]. In this process, it is important to mention that the remodeling of cristae structures at the IMM has been demonstrated to facilitate the release of cytochrome c [63]. Interestingly, a recent study has found that cytochrome c can induce CL peroxidation independent of ROS, which is controlled by its redox states [63]. The authors hypothesize that a two-step process (the ROS-independent and ROS-dependent steps) might be involved in the early stage of apoptosis before cytochrome c release [63], although further studies are required to demonstrate this statement.

**Figure 3:**
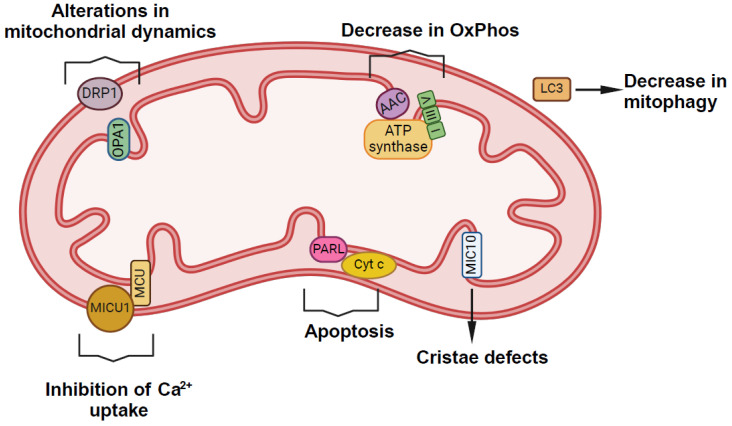
Schematic representation of proteins and biological processes affected by CL alterations within mitochondria. Abbreviations: AAC, ADP/ATP carrier; I, III, IV, complex I, III, IV (respectively); MUC, mitochondrial Ca^2+^ uniporter; MICU1, mitochondrial calcium uptake 1; OPA1, OXPHOS, oxidative phosphorylation; PARL, presenilin-associated rhomboid-like protein; DRP1; dynamin-related protein 1; OPA1, optic atrophy 1; LC3, microtubule-associated protein 1A/1B-light chain 3; Cyt c, cytochrome c; MIC10, mitochondrial contact site and cristae organizing system 10.

The oxidation of CL leads to conformational changes that affect the physical properties of the IMM and OXPHOS activity [64]. Oxidized CL acts as a signaling molecule to promote mitophagy (mitochondrial degradation) and/or apoptosis (programmed cell death). During mitophagy, CL traverses the IMM to arrive at the OMM in a process facilitated by the mitochondrial nucleoside diphosphokinase [65] and/or phospholipid scramblase 3 [66]. Once externalized, CL recruits microtubule-associated protein 1A/1B-light chain (LC3) [66]. The N-terminal variability of the LC3 protein subfamily determines its specificity for CL. Thus, the LC3A (MAP1LC3A) exhibits a high affinity and specificity for CL, actively participating in CL-mediated mitophagy [67]. LC3C (MAP1LC3C) has the highest affinity for CL but does not partake in CL-mediated mitophagy. Instead, it is proposed that LC3C’s binding to PA on the OMM contributes to maintaining the integrity of the mitochondrial network under normal growth conditions through the selective degradation of mitochondrial proteins [67]. During apoptosis, the externalization of oxidized CL promotes the increase in caspase-3 activity and Bcl-2–associated X protein (Bax) [67,68]. Therefore, alterations in the CL profile result in the malfunction of proteins and enzymes, leading to mitochondrial dysfunction and disease. Relevant proteins affected by CL dysfunction leading to mitochondrial alterations are MIC10 subunit [29,30,31], F_1_F_o_ ATP synthase [28,69], RCs [70], ADP/ATP carrier [39], MCU [41,42], PARL [71], DRP1 [47] [72], OPA1 [48], LC3 [67] and cytochrome c [73]. A full list of proteins affected by CL dysfunction, alongwith their corresponding mitochondrial alterations in each biological model, is summarized in Table 1 and represented in Figure 3.

## 5. The Role of Cardiolipin in Neurodegenerative Diseases

The brain exhibits a CL profile with the highest molecular diversity, comprising up to 100 different species [6,74,75]. This extraordinary diversification may be attributed to its various functions. It is believed that the role of CL in structural organization is facilitated by the presence of non-PUFA species (with saturated and monounsaturated acyls) and symmetric linoleoyl-containing CL species, thus leading to its predominant presence in mammalian tissues with high bioenergetic demands, such as the heart and skeletal muscles. Conversely, the role of CL in the signaling function is thought to be carried out by longer-chain PUFA-containing species, as found in the brain [74,76,77]. At the cellular level, it has been found that the content of CL is roughly two-fold higher in glial cells compared to neurons [78]. This difference in CL levels may contribute to variations in intracellular signaling molecules and functional activity between these two primary types of brain cells [78]. Interestingly, CL content is reduced in non-synaptic mitochondria compared to synaptic mitochondria from the brains of aged rats [79]. The distinct mechanisms by which the tissue- and cell-specificity of CL compositions are generated and regulated are still largely elusive.

Given that the brain consumes over 20% of the total body energy, CL abnormalities, including changes in the CL content, FA acyl chain composition, and CL oxidation in the CNS, are unsurprisingly linked to various neurodegenerative diseases. Consequently, gaining a comprehensive understanding of the role of defective CL metabolism in CNS homeostasis and brain function is likely to provide significant insights into the pathophysiology of neurodegenerative processes. The recent findings in CL-mediated neurodegenerative disorders are summarized as follows (see Table 2).

### 5.1. Alzheimer’s Disease (AD)

AD is the most prevalent adult neurodegenerative disorder. Pathologically, it manifests through progressive neuronal loss in the hippocampus and cortex, accompanied by the accumulation of extracellular neuritic plaques and intracellular neurofibrillary tangles in the brain. A prominent component within these plaques is β-amyloid (Aβ), formed through the cleavage of the amyloid precursor protein (APP) by presenilin-1 (PS1) and/or presenilin-2 (PS2), both integral to the γ-secretase complex [94]. Significantly, dominantly inherited mutations in both presenilins and APP currently represent the sole known causes of familial Alzheimer’s disease (FAD). This observation has led to the widely accepted “amyloid cascade” hypothesis, positing that the deposition of Aβ in the brain serves as the initiating pathological event in AD [95]. While this hypothesis elucidates the development of plaques and, potentially, tangles, it fails to explain other facets of the disease [96]. Recent studies propose an alternative perspective, suggesting that AD may be rooted in a disorder of lipid metabolism [97,98,99]. A low CL content associated with impaired mitochondrial synaptic function and oxidative stress has been reported in vivo [11], and in vitro [80]. In addition, a significant reduction of CL that contained polyunsaturated FA (PUFA) was observed in the cortex of human brains affected by AD [81]. Both in vivo and in vitro models of AD have demonstrated the high susceptibility of mitochondrial membranes to tau aggregates, resulting in the overexpression of tau aggregates, resulting in neuronal toxicity [100]. Tau protein exhibits a preference for binding to CL-rich regions of the OMM, triggering mitochondrial swelling, cytochrome c release, and a reduction in membrane potential in vitro [82]. Another aspect of AD is the dysregulated neuroinflammatory response, caused by persistent microglial activation and the release of proinflammatory cytokines. A potential protective role of CL in mitigating the AD inflammatory response has been postulated [83]. Finally, it has been demonstrated that alterations in the ATP-binding cassette (ABC) subfamily A member 7 gene (ABCA7), identified as a strong risk factor for late-onset AD, induce a reduction in CL content [84]. While recent advances in lipidomics have proposed other lipids as potential biomarkers in AD using blood samples from living patients and brain tissue from autopsy patients (reviewed by [101,102]), there have been no clinical studies evaluating the levels of CL in AD patients to date.

### 5.2. Parkinson’s Disease (PD)

PD is the most prevalent movement disorder and the second most common neurodegenerative disease after AD. Pathologically, PD is caused by the loss of dopaminergic neurons in the substantia nigra, a basal ganglia structure in the midbrain, which affects both cognitive function and gait. It is one of the synucleinopathies diseases, whose hallmark is represented by Lewy bodies, which are characterized by alpha-synuclein (α-syn) inclusions [103]. The protein α-syn is intrinsically disordered and it is highly expressed in neurons [104]. The majority of PD is idiopathic, with familial PD constituting 5–10% of cases. Recent data suggest that α-syn interacts with CL, potentially impacting the mitochondrial membrane integrity [85,105]. However, the pathomechanism behind this interaction is still unclear. OMM-localized CL promotes the refolding of disordered α-syn monomers to an α-helical structure, preventing neuronal loss and leading to mitophagy [23]. Moreover, α-syn can form a triple complex with CL and cytochrome c, which prevents cytochrome c release and delays neuronal damage [106]. This observation is in contrast to reports that anionic membranes promote α-syn aggregation [107]. Indirect evidence also suggests that α-syn oligomers interact with CL to favor pore formation within mitochondrial membranes affecting membrane permeability [86]. The formation of α-syn fibrils increased in the presence of CL leading to the unfolded protein response (UPR) in the ER and mitochondria [87]. Interestingly, the localization of α- syn at ER–mitochondrial contact sites was also reported in different models [108,109]. These studies have shown that the overexpression of α-syn disrupted the physical contacts and Ca^2+^ transfer between the two organelles, leading to alterations in mitochondrial metabolism. It is possible that aggregates of α-syn could, thus, directly disrupt mitochondrial metabolism via interactions with CL-rich microdomains or contact sites between the mitochondria and ER. The inhibition of the enzyme ALCAT1 may reduce α-syn oligomerization, preventing apoptosis [88]. On the other hand, no changes in the total CL content have been observed across various PD murine models [12] or PD patients [89]. A lipidomic analysis in rat rotenone models of PD revealed a notable loss of linoleic-acid-containing CL species and the accumulation of CL oxidation in the substantia nigra during later stages of exposure. In contrast, elevated levels of PUFA-containing CLs were detected in plasma, suggesting that non-apoptotic cell death pathways are also triggered in rotenone-treated animals [16]. These collective findings underscore the significant role of CL in PD, raising the possibility that CL species might be important biomarkers in neuropathological stages [101]. Whereas new lipidomic analysis using whole blood and serum from PD patients reveals that the progression of the disease could be predicted by a lipid-biomarker panel [110,111,112], CL is not yet included in those studies, similar to what we found in AD.

### 5.3. Amyotrophic Lateral Sclerosis (ALS)

ALS is a fatal neurological disorder characterized by the selective loss of motor neurons, resulting in muscle atrophy, paralysis, and respiratory failure. The exact cause of ALS remains uncertain, with around 90% of cases having unknown origins (termed as sporadic ALS), and the remaining 10% identified as hereditary cases (known as familial ALS). For familial ALS, 42 genes including SOD1 (superoxide dismutase 1), C9ORF72 (chromosome 9 open reading frame 72), TARDBP (tAR DNA-binding protein), and FUS (fus RNA-binding protein) have been identified to be causal factors [113]. Interestingly, there is growing evidence linking alterations in lipid metabolism to ALS pathogenesis [114], although its implications remain unknown. The mouse model of ALS with FUS overexpression exhibits high levels of cholesterol esters, specific ceramides, and the dysregulation of phospholipids, including reduced levels of cardiolipin [13]. Along this line, the reduction of CL levels in the spinal cord has also been observed in the transgenic rat model SOD1-G86R (superoxide dismutase 1 Gly86Arg), along with other lipidomic changes [14]. Notably, the spinal cord and, to a lesser extent, brain mitochondria of SOD1-G93A transgenic mice exhibit increased CL peroxidation, impaired OXPHOS activity, and heightened cytochrome c release from the IMM, aligning with the low binding affinity of oxidized CL for cytochrome c [115]. A recent study has shown no significant changes in CL in the serum from ALS patients compared to controls, but the authors observed an increase in the unsaturated FA, which may affect the different species of CL [90]. These emerging lines of evidence suggest that aberrant CL metabolism plays a broader role in ALS pathogenesis. Future studies will help to understand better the association between CL and ALS.

### 5.4. Charcot–Marie–Tooth Type 2B (CMT2B)

CMT2B is a rare hereditary peripheral neuropathy resulting from five missense mutations in the RAB7A (ras-related protein Rab-7a) gene, responsible for encoding a small GTPase within the RAB (ras-related in the brain) family. [116]. Currently, no cure is available for this disease. Pathologically, the CMT2B peripheral neuropathy is characterized by prominent sensory loss, progressive distal weakness leading to atrophy, reduced tendon reflexes, and normal or near-normal nerve conduction [116]. Notably, a recent lipidomic analysis has revealed changes in the lipidomic profile of CMT2B-derived fibroblasts versus healthy donor cells. The authors found that CMT2B cells exhibited an elevated level of monounsaturated FA and increased expression of key enzymes involved in monounsaturated and polyunsaturated FA synthesis [91]. Although the authors did not include the analysis of CL, it is conceivable that its content is also altered.

### 5.5. Traumatic Brain Injury

Traumatic brain injury (TBI) results from a sudden, external force causing damage to the brain. The severity of TBI can range from acute to chronic, leading to cognitive, emotional, and physical impairments. In fact, TBI is a leading cause of neurodegenerative diseases. Recent studies using mass spectrometric imaging have revealed that early phases of TBI exhibited reductions in CL levels in the contusional cortex, ipsilateral hippocampus, and thalamus, with the most highly unsaturated CL species being particularly vulnerable to loss [77]. Moreover, TBI is associated with an increase in mitophagy leading to the externalization of CL to the OMM, thereby eliminating mitochondrial damage and preventing neuronal death [92]. Interestingly, recent studies are investigating the detection of CL in animal and human biofluids like blood, urine, saliva, and tears for its potential role as a biomarker in diagnosing traumatic brain injury (TBI) [15,117].

### 5.6. Spinal Cord Injury

Traumatic spinal cord injury (SCI) results in neurological deficits below the level of injury. Currently, there is no effective treatment available for SCI patients. A recent research study has revealed a significant 50% decrease in CL species identified in adult rat spinal cord following a moderate contusive SCI [93]. The decreased CL species predominantly contained polyunsaturated fatty acids, making them highly susceptible to peroxidation. The authors also found that mitochondrial oxidative stress induced CL oxidation and led to CL loss by activating iPLA2γ to hydrolyze CL. These CL alterations induced mitochondrial dysfunction and subsequent neuronal death. Remarkably, simultaneous measurements of cytochrome c release, apoptotic protein Smac/DIABLO (second mitochondria-derived activator of caspases/direct IAP binding protein with low pI) release, and caspase-3 activity were used in this study as biomarkers to assess the extent of cardiolipin loss in rat models of SCI [93].

## 6. Cardiolipin-Based Therapeutics

The identification and characterization of novel CL-binder candidates that have the potential to attenuate mitochondrial damage is a priority. SS-31 (Szeto-Schiller-31), or elamipretide, has been proposed as a first-in-class group of compounds capable of restoring cellular bioenergetics. Elamipretide prevents the conversion of cytochrome c into a peroxidase by interacting with CL, thereby preserving the mitochondrial membrane integrity and enhancing OXPHOS [118,119]. The positive effects of this agent have been demonstrated in models of AD [120,121,122], PD [123], and peripheral mitochondrial injury diseases [124]. A Phase III trial assessing elamipretide in models of mitochondrial myopathy failed to achieve its primary endpoints [125]. One reason is the relatively weak interaction of the SS peptide against CL [126]. Alternatively, a closely related compound to SS-31, known as SBT-272 (bevemipretide trihydrochloride), has been identified. This drug is a novel peptidomimetic under development by Stealth BioTherapeutics Inc. The mechanism of action is similar to SS-31; however, SBT-272 shows a higher mitochondrial uptake, greater concentrations in the brain, and a higher bioavailability [127]. Importantly, SBT-272 counteracts the proteinopathy TDP-43 (TAR DNA-binding protein 43) in ALS upper motor neurons by modulating mitochondrial integrity, motility, and function [127]. SBT-272 is currently in a Phase 1 trial in healthy subjects.

Aside from them, new promising discoveries of CL-targeting peptides have recently been proposed. For instance, the synthetic CMP3013, a cyclohexylalanine-containing α-helical amphipathic peptide, has been demonstrated as a protective agent preserving the mitochondrial cristae structure and enhancing ATP production in models of kidney dysfunction. CMP3013 exhibits high selectivity for CL compared to other IMM lipid components [128]. Remarkably, the pharmacologic inhibition of CL alterations with XJB-5-131, a synthetic mitochondria-targeted electron and reactive oxygen species scavenger, attenuated cell death and tissue damage, and ameliorated motor deficits after SCI in adult rats [93,129]. Notably, supplementation with eicosapentaenoic acid (EPA) from fish oil has been demonstrated to elevate the mitochondrial membrane potential and CL levels in astrocytes. However, it does not alter ATP levels, implying that its utilization may help to prevent neurodegenerative diseases [130]. A recent study also suggests that the bacterial probiotic *Weizmannia coagulans* lilac-01 (Lilac-01EVs), characterized by a high concentration of CL and PG in the cell membrane, may have the ability to inhibit cell death in primary microglia [131].

Lastly, using computational screening methods, a recent study has discovered a new drug called CardioLipin-Binder (CLiB) that specifically targets CL [132]. This study showed that CLiB increased the respiration of CL-containing intact bacterial cells and isolated mitochondria. Hence, CLiB may serve both basic research and, potentially, therapeutic purposes. Overall, these drugs represent a potential target for pharmacological strategies aimed at treating neurodegeneration. Further research has to be carried out in order to prove their benefits in clinical trials.

## 7. Conclusions and Perspectives

In the last few years, clinical and experimental studies from human and animal models have demonstrated evidence linking aberrant CL metabolism with neurological dysfunction, suggesting that CL may serve as a biomarker candidate for neurodegenerative diseases. However, the specific physiological roles of CL in different cell types and its contribution to neurodegeneration are not fully understood. Future studies should explore the cell-type-specific aspects of CL biosynthesis and remodeling. Moreover, since brain biopsy samples of living patients are not available, alternative samples such as blood, urine, or saliva should be employed in lipidomic studies to identify “CL signatures” that predict the severity of the disease. Recent discoveries in new drugs and dietary supplements that target CL by regulating its availability in cells should also be studied in human clinical trials for therapeutic purposes. Adopting innovative, multidisciplinary approaches will be essential for a comprehensive understanding of these roles and their impact on nervous system homeostasis and brain function.

## Figures and Tables

**Table 1:** List of proteins affected by CL dysfunction.

Protein Affected	Mitochondrial Alterations	Biological Sample	Ref.
MIC10 subunit	-Cristae defects -Misdistribution of MRC	-Yeast -Hela, HepG2 cells	[29,30,31]
F_1_F_o_ ATP synthase	-Non-bilayer structure formation -Decrease dimerization ATP synthase in the cristae -Reduce OXPHOS	-Synthetic liposomes -Drosophila	[28,69]
-RCs (I, III) -RSCs (I, III, IV)	-Reduction in RCs -Misfold of RSCs	-Mouse	[70]
ADP/ATP carrier (AAC)	-Destabilized protein structure -OXPHOS defects.	-Yeast -Human cell lines	[39]
MCU, MICU1	-Reduced mitochondrial Ca^2+^ uptake -Inactivation of pyruvate dehydrogenase	-BTHS patient cells and cardiac tissue	[41,42]
PARL	-Increase the expression of PARL leading to apoptosis	HEK293 cells	[71]
DRP1	-Reduce mitochondrial fission	-Synthetic liposomes	[47]
p-ser579-DRP1 p-ser600-DRP1	-Reduce DRP1 activation	-Synthetic liposomes	[72]
OPA1	-Alterations mitochondrial fusion	-Synthetic liposomes	[48]
LC3	-Decrease mitophagy	-Synthetic liposomes -SH-SY5Y cells	[67]
Cytochrome c	-Apoptosis	-HL-60 cells	[73]

Abbreviations: BTHS, Barth syndrome; CL, cardiolipin; MIC, mitochondrial contact site and cristae organizing system; MUC, mitochondrial calcium uniporter; MICU, mitochondrial calcium uptake 1; RSCs; respiratory supercomplexes; RCs respiratory complexes; OXPHOS, oxidative phosphorylation; PARL, presenilin-associated rhomboid-like protein; DRP1; dynamin-related protein 1; OPA1, optic atrophy 1; LC3, microtubule-associated protein 1A/1B-light chain 3.

**Table 2:** Recent findings in CL-associated neurodegenerative diseases.

Disease	Model	Findings	References
**AD**	Brain from 3xTg-AD mice	-Reduced CL species in synaptic mitochondria -Lack of detection of oxidated CL	[11]
	SH-SY5Y-APPswedish	-Decrease in total CL content -Alterations in PG	[80]
	Brain from AD patients	-Slightly reduction of CL content -Decrease in FA content	[81]
	SH-SY5Y	-Tau protein exhibits a preference for binding to CL-rich regions of the OMM	[82]
	Primary microglia cultures and neuron cell lines	-CL inhibits amyloid-β secretion	[83]
	-Human iPSC ABCA7-KO -Brain from Abca7-KO mice	-Reduction of CL content -Decrease ATP synthesis, increase in ROS, and increase mitochondrial fusion	[84]
**PD**	SH-SY5Y	-CL accelerates the rate of α-synuclein fibrillization, leading to hyperactive respiration	[85]
	SNCA-mutant human pluripotent stem cells (iPSCs) and SNCA-transgenic mice	-Exposed CL to the OMM binds to and facilitates refolding of α-syn fibril. -Prolonged CL exposure in SNCA-mutants initiates recruitment of LC3 to the mitochondria and mitophagy.	[23]
	Freshly isolated mitochondria or liposome	-CL interacts with α-syn to favor pore formation within mitochondrial membranes	[86]
	N27 rat dopaminergic cell line	-CL increase α-synuclein aggregation, leading to ER stress	[87]
	Brain from MPTP mouse	-Inhibition of ALCAT1 prevents neurotoxicity, apoptosis, and motor deficiencies.	[88]
	Brain from Parkin-KO mice	-Lack of changes in CL content -CL remodeling defects with increase of short, saturated CL acyl-chains	[12,89]
	Brain from rat rotenone model	-Exposure to rotenone induces a loss in linoleic acid-containing CL species and an increase in CL oxidation	[16]
**ALS**	Spinal cord from FUS mice	-Reduction in CL content	[13]
	Cortex and spinal cord from SOD1-G86R mouse	-Reduction in CL content	[14]
	Serum ALS patients	-No changes in CL content -Increase in unsaturated lipids	[90]
**CMT2B**	Fibroblasts from CMT2B patient	-Lack of measure of CL content by lipidomics -Increase in levels of unsaturated FA	[91]
**TBI**	Brain from TBI rat model	-Reduction of CL content	[77]
	Brain from TBI patients and TBI rat models	-CL drives mitophagy	[92]
**SCI**	Spine from SCI rat models	-Decrease in CL content and increase in CL oxidation	[93]

Abbreviations: CL, cardiolipin; Tg, transgenic, AD, Alzheimer’s disease; APP, amyloid precursor protein; PG, phosphatidylglycerol; FA, fatty acids; OMM, outer mitochondrial membrane; iPSC, induced pluripotent stem cells; ABCA ATP-binding cassette sub-family A member; ROS, reactive oxygen species; PD, Parkinson’s disease, SNCA, synuclein alpha; KO, knockout; LC3, microtubule-associated protein 1A/1B-light chain 3; ER, endoplasmic reticulum; MPTP, methyl-4-phenyl-1,2,3,6-tetrahydropyridine; ALCAT1, acyl-CoA:lysocardiolipin acyltransferase-1; ALS, amyotrophic lateral sclerosis; FUS, FUS RNA-binding protein; SOD, superoxide dismutase 1; CMT2B, Charcot–Marie–Tooth Type 2B; TBI, traumatic brain injury; SCI, spinal cord injury.
